# Attacking the Achilles heel of cardiac amyloid nuclear scintigraphy: How to reduce equivocal and false positive studies

**DOI:** 10.1007/s12350-023-03214-6

**Published:** 2023-03-01

**Authors:** Zainab Al Taha, Deniz Alibazoglu, Hani Sabbour, Ingy Romany, Haluk Alibazoglu, Sabahat Bokhari

**Affiliations:** 1 Sheikh Shakhboot Medical City, Abu Dhabi, United Arab Emirates; 2 Northwestern University, Chicago, IL USA; 3 Cleveland Clinic Abu Dhabi, Abu Dhabi, United Arab Emirates; 4 Warren Alpert School of Medicine, Brown University, Providence, RI USA; 5 Pfizer Gulf FZ-LLC, Dubai, United Arab Emirates; 6 Cleveland Clinic Abu Dhabi, Abu Dhabi, United Arab Emirates; 7 Robert Wood Johnson Medical School – Rutgers University, New Brunswick, NJ USA

**Keywords:** Amyloid heart disease, CT, SPECT, Image Reconstruction, Technical

## Abstract

**Background:**

Planar and single-photon emission computed tomography (SPECT) nuclear imaging techniques with bone seeking radiotracers have been increasingly adopted for diagnosis of ATTR cardiac amyloidosis. However, inherent limitations of these techniques due to lack of anatomical landmarks have been recognized, with consequent high numbers of equivocal or false positive cases. SPECT/computed tomography (CT) fusion imaging offers a significant advantage to overcome these limitations by substantially reducing inaccurate interpretations. The authors present the results of a 3-year imaging quality improvement project that focused on reducing the high number of equivocal studies that were noted in the first two years of the amyloidosis program, comparing SPECT only to SPECT/CT fusion technique.

**Methods:**

A retrospective, systematic analysis of 176 patient records was performed to test the premise that SPECT/CT fusion imaging has the potential to reduce equivocal and false positive results.

**Results:**

Of a total of 176 patients, 35 equivocal (19.8%), 32 (18.18%) strongly suggestive, and 109 (61.93%) not suggestive cases were identified. Recognizing that this was not consistent with the international data, the authors set out on a comprehensive quality assessment project to reduce the number of equivocal and false positive cases. In patients who initially underwent SPECT only (Group A; *n* = 78), the addition of SPECT/CT fusion resulted in the net reclassification of 73% of cases: 100% of equivocal cases (*n* = 35) were reclassified to not suggestive (*n* = 34) or strongly suggestive (*n* = 1). 73% of strongly suggestive cases (*n* = 30) were reclassified to not suggestive (*n* = 22) while 8 strongly suggestive cases were confirmed as true positives. 13 not suggestive cases remained negative after SPECT/CT fusion. In cases where SPECT/CT fusion was utilized from the beginning (Group B; *n* = 98), there were no reclassification of any of the cases when these cases were reprocessed as a control group.

**Conclusion:**

Addition of SPECT/CT imaging reduces the false positive or equivocal studies and increases the diagnostic accuracy of the test. All false positive and equivocal studies were eliminated using the fusion technique. Utilizing the fusion imaging technique increases the spatial resolution, with the ability to localize myocardial uptake and accurately differentiate from blood pool, which is a major source of error.

**Supplementary Information:**

The online version contains supplementary material available at 10.1007/s12350-023-03214-6.

## Introduction

The widespread availability of systemic evaluation of the scintigraphy with phosphate tracers has led to increased adoption of Tc 99m-pyrophosphate (PYP) imaging in screening for cardiac amyloidosis, with a subsequent increase in identifying patients with the disease.^1^ However, there are potential pitfalls in image processing and interpretation that can lead to incorrect diagnosis. Single-photon emission computed tomography (SPECT) provides a method for improved assessment of amyloid burden adjunct to the visual semi-quantitative interpretation of planar images^2^ and the ASNC Expert Consensus recommends SPECT to be performed in all amyloid imaging studies in order to confirm myocardial uptake and differentiate it from blood pool uptake.^3^

However, the Achilles heel of planar and conventional SPECT imaging is excessive blood pool uptake in the left ventricular (LV) cavity which impairs the ability to truly delineate the tracer uptake in myocardial wall and results in inaccurate display of images that suggests myocardial uptake, invariably leading to high percentages of false positive or equivocal studies. As the non-invasive diagnosis of ATTR-CM relies entirely on the tracer uptake within the myocardium, this is a critical point to resolve in laboratories starting to undertake these imaging studies.

Hybrid cardiac imaging with SPECT and computed tomography (CT) offers improved diagnostic information compared with planar and SPECT imaging.^4^ While the 2019 ASNC guidelines^3^ and the Addendum published in 2021^5^ recommends SPECT for all amyloid imaging studies, SPECT/CT fusion is only recommended for ancillary findings, and not for localization of the myocardium or blood pool.

The Cardiac Amyloidosis program at Cleveland Clinic Abu Dhabi (CCAD) is an example of a nascent amyloidosis program in the Middle East-Gulf region that utilizes the SPECT/CT fusion technique as a standard practice in screening for cardiac amyloidosis.

## Evolution of planar imaging, visual grading, H/CL ratio, and SPECT only imaging to SPECT/CT fusion

Between 2018 and 2019, it was a standard practice at CCAD to use planar imaging, visual grading, H/CL ratio, and SPECT imaging in screening for cardiac amyloidosis. However, with increasing imaging volumes and interpretation experience, the number of equivocal and strongly suggestive results were disproportionately high compared to the number of not suggestive results. The protocol for patients with equivocal results was to reimage after 6–12 months. The rationale for re-scanning equivocal cases after six months, was to identify early disease in these patients and to initiate treatment as soon as possible.

By late 2019, CT was added to our image acquisition; however, as CT was not yet implemented in the guidelines and consensus recommendation at that time,^3^ attention was not focused on CT fusion. As such, SPECT and CT images were not properly fused and post-processed, and therefore not incorporated in our routine reporting system. H/CL ratio and visual score was the gold standard for diagnosing patients in our center, which was in line with the guidelines and consensus recommendations at that time.

However, we came to realize that the number of equivocal cases for our center (based on H/CL ratio and visual score) exceeded the numbers reported in literature (45% *vs* 35% in AL amyloid, 19% in no amyloid (not suggestive) and 2% in ATTR (positive)^6^ or 23% (with SPECT only).^7^ It is important to note, however, that Tamarappoo et al.^6^ suggested the use of dual energy thallium (1 mci) acquisition to improve the localization of PYP in the myocardium, which exposes patients to a much higher radiation burden than CT.

**Figure 1:**
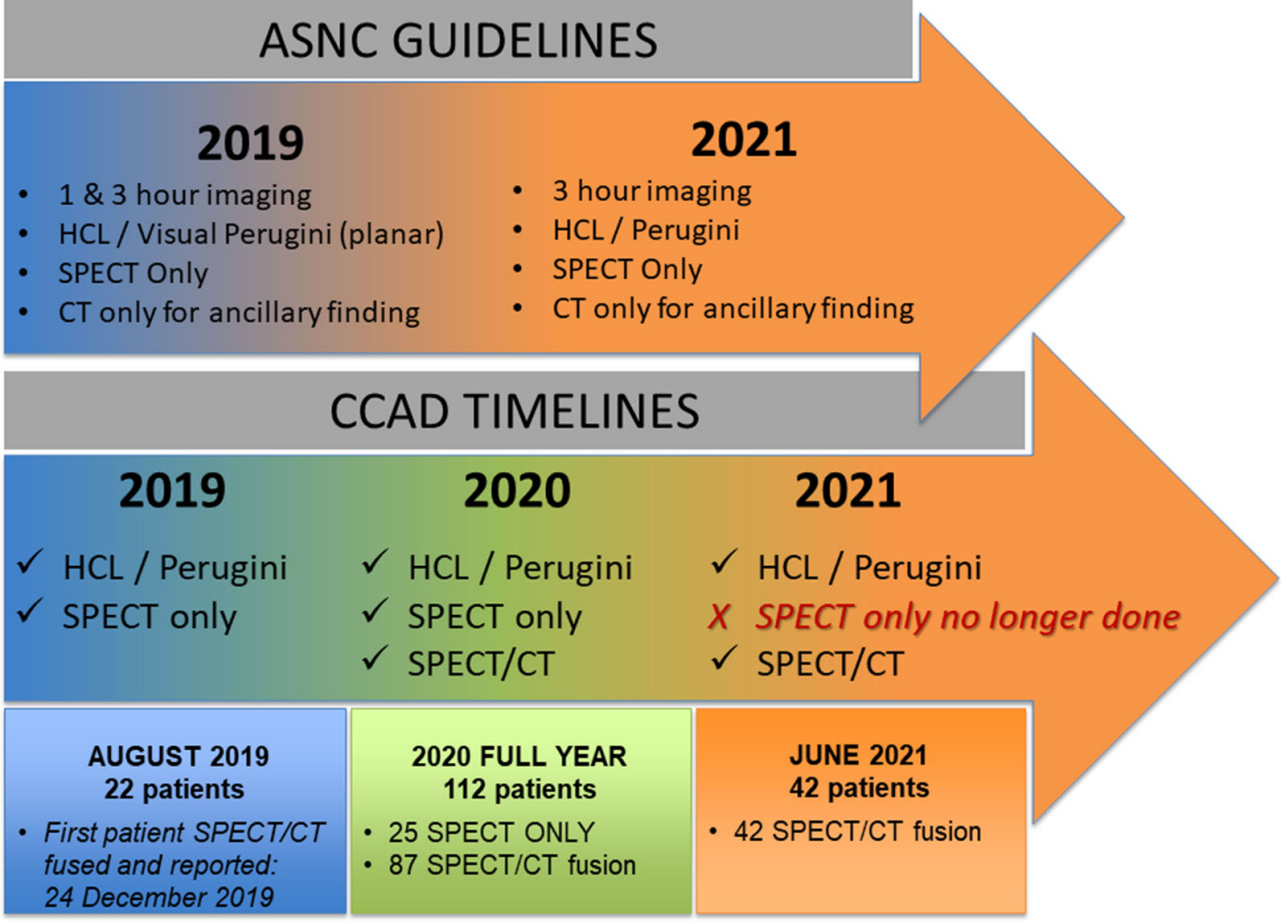
Timeline of cardiac amyloidosis program at CCAD

Therefore, in early 2020, CT was added to SPECT as a way of determining whether SPECT/CT provides improved diagnostic accuracy. This fusion technique resulted in the elimination of equivocal and false positive results in most of the cases. Once SPECT/CT fusion was recognized to have additional diagnostic value, this technique was adopted as the standard practice for cardiac amyloidosis at CCAD and SPECT and CT images were acquired for all subsequent patients (Figure 1).

However, there were initially certain processing and misregistration errors which contributed to suboptimal post-processing of the images. Realizing the learning curve of this new technique, a systematic reanalysis of the images was performed, comparing SPECT with SPECT/CT fusion. Through accurate manual co-registration of SPECT and CT data, equivocal results were eliminated, and many strongly suggestive results were reclassified as not suggestive.

The authors formulated a hypothesis that SPECT/CT fusion eliminates equivocal and false positive results in a great majority of cases. This hypothesis was tested by systematically and manually applying this technique to the entire cohort. The insights gained by continuous quality improvement and reanalysis, have identified common pitfalls in the image interpretation process. Considering that PYP scan is currently the gold standard of non-invasive diagnosis of ATTR cardiac amyloidosis, imaging physicians should consider these pitfalls in image interpretation.

In this paper, the authors share their experience of reclassification of final image interpretation through utilization of the SPECT/CT fusion technique, highlighting potential pitfalls that may lead to erroneous results if SPECT only imaging is utilized.

The objective of this paper is to highlight the significant impact of the SPECT/CT fusion technique in improving precision in diagnosis by truly identifying tracer uptake within the LV myocardium and tracer pooling within the LV cavity. This paper also highlights the importance of accurate post-processing of SPECT/CT images in order to achieve the best outcome from the SPECT/CT technique, which leads to complete reclassification of the initial interpretation.

## Methods

A retrospective, systematic analysis of the CCAD cardiac amyloidosis patient database was initiated in 2019 to test the hypothesis that SPECT/CT fusion has the potential to eliminate equivocal and false positive results. All patients were imaged on a Siemens Symbia T SPECT/CT scanner.

The retrospective re-analysis covered patient records for the period between August 2019 to June 2021 and included 176 patients who initially underwent PYP imaging for the detection of cardiac amyloidosis. PYP findings, ECHO findings, free light chain assays, serum and urine electrophoresis for monoclonal proteins with immunofixation, magnetic resonance imaging (MRI) findings, demographics and clinical findings for all patients were collected. Over the period of three years all 176 patients were included in the systematic reanalysis using H/CL ratio, visual grading (Perugini grade) and SPECT, and later SPECT/CT fusion.

The study cohort was split into two groups: Group A included 78 cases where CT was not used to confirm the diagnosis. Planar (H/CL ratio and visual score) and SPECT was used for diagnosis in this group. Of these 78 patients, 43 patients had previously acquired CT scans; although the routine acquisition of CT dataset was not being used for SPECT/CT fusion at this time, but only utilized for ancillary findings as per the ASNC guidelines.^3^ The remaining 35 patients in Group A did not have CT as part of the initial image acquisition. These patients were brought in for a repeat scan with CT fusion. These 35 patients underwent repeat study at the early stage of the learning curve for our center. Due to the high index of clinical suspicion (red flags), we were concerned that this may represent an early stage of the disease or technical issues, and in order to closely follow up our patients, a repeat study was scheduled at 6–12 months, which ultimately did reflect in the reclassification of these patients. The repeat study used SPECT/CT fusion. The interval period of repeating the scan was on average 6–12 months (8.9 ± 0.4 months). For the remaining 43 patients, retrospective fusion was performed. As part of the quality improvement project, each of these cases were manually processed with SPECT/CT fusion (retrospectively) with specific attention to the localization of the anatomical structures by careful fusion of the CT on the SPECT, to ensure the myocardium and blood pool differentiation. Group B consisted of 98 cases that had SPECT/CT fusion done properly from the beginning (starting in 2020). All of these studies were reprocessed and reread as well and served as the control arm.

In order to reduce bias from interpersonal variability, all studies were read by a single interpreter (experienced CBNC and ABNM certified nuclear radiologist) for both the initial interpretation and the reanalysis and reclassification. The expert physician performed the manual post-processing and fusion himself and was blinded to the clinical attributes of the patients. In addition, the images were systematically reviewed in a weekly multidisciplinary team (MDT) imaging meeting. The MDT consists of three CBNC certified cardiologists, two cardiac radiologists CBNC / ABR, and one CBNC & ABNM certified nuclear cardiologist. Details on reconstruction and post-processing of the images are presented in Appendix A.

## Results

Out of the total cohort of 176 patients, there were initially 35 (19.8%) equivocal, 32 (18.18%) strongly suggestive, and 109 (61.93%) not suggestive cases. In group A (*n* = 78; where planar and SPECT only was used as the gold standard), 35 (44.8%) cases were equivocal, and 30 (38.4%) cases were strongly suggestive; even though the imaging appeared to be largely blood pool.

After application of SPECT/CT to this patient pool—either by repeat study with SPECT/CT fusion or reprocessing the initial study with previously acquired CT—100% of equivocal cases were reclassified (34/35 to not suggestive and 1/35 to strongly suggestive). Of the 30 strongly suggestive cases 22 (73%) were reclassified to not suggestive and 8 (27%) true positive (strongly suggestive) cases were confirmed. For the not suggestive cases there were no confounding blood pool activity and these cases remained negative even after CT fusion; confirming that blood pool activity is a major confounder and highlighting the need to differentiate the myocardium from the LV or right ventricle (RV) blood pool.

**Figure 2:**
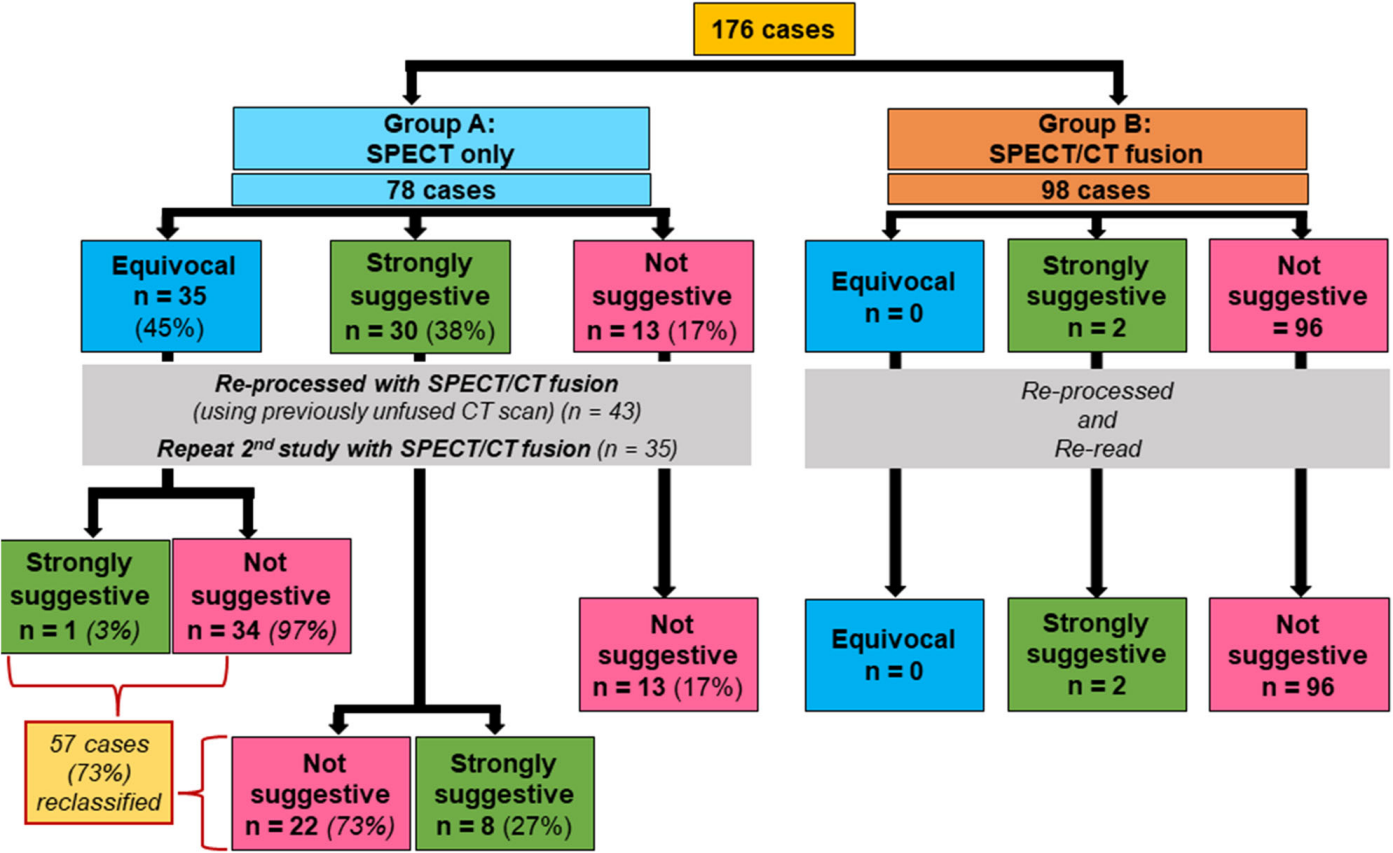
Total cohort: 176. Group A: 78 cases that had SPECT only in the beginning. After reprocessing with SPECT/CT fusion, a total of 57 cases were reclassified; 35 equivocal cases were reclassified as either strongly suggestive (*n* = 1) or not suggestive (*n* = 34) and 22 strongly suggestive cases were reclassified as not suggestive (false positives). Group B: 98 cases that had SPECT/CT fusion done properly from the beginning, being the more advanced technique; resulted in zero cases being reclassified during the “re-analysis”; served as the control arm

This is in stark contrast to group B, where 98 patients underwent SPECT/CT fusion in the initial study, as part of our standard workflow. When these cases were reprocessed and reread as part of the quality improvement project, there were no reclassification of any of the cases, i.e., 100% of cases were correctly classified either as strongly suggestive (*n* = 2; 2%) or not suggestive (*n* = 96; 98%) from the start utilizing the SPECT/CT fusion technique. There were no equivocal cases in this group (Figure 2). As a result of this learning, we have been performing SPECT/CT fusion as the standard workflow in our center since 2021 (total number of 177 cases since January 2021).

The data therefore show a Net Reclassification Improvement of diagnostic accuracy of PYP imaging of 73% when SPECT/CT is added. This improvement was dependent not only on the SPECT/CT fusion technique, but also accurate image post-processing of the acquired SPECT/CT data. By utilizing this technique, the specificity and sensitivity of the testing was refined and improved substantially compared to using SPECT alone.

## Discussion

Although SPECT imaging is required for the diagnosis of cardiac amyloidosis, SPECT/CT imaging has always been optional and only included in the addendum for multi-society Expert Consensus Recommendations for Multimodality Imaging in Cardiac Amyloidosis.^5^

Given the significant learning curve involved in image interpretation, our experience demonstrated that it might not be possible to accurately classify all cases with planar and SPECT only imaging. The greatest difficulty in SPECT only imaging is to define the interventricular septum versus the LV blood pool, which is the main culprit for equivocal and/or false positive interpretations. Distinguishing the LV cavity from the LV septum can be challenging due to reduced spatial resolution, the inherent limitation of SPECT imaging. With the introduction of additional CT imaging to delineate anatomical landmarks, precise identification of the myocardial borders affords accurate differentiation of tracer activity in LV cavity and true LV myocardial uptake.

The authors found that not only did SPECT/CT fusion reduce equivocal cases, but it also reduced false positive cases. Based on this analysis, applying SPECT/CT fusion as part of standard practice significantly impacted on the final diagnosis of patients suspected to have cardiac amyloidosis and resulted in the net reclassification of 73% of cases. Fusing SPECT and CT allows for highly robust differentiation of the truly positive scans from false positive and equivocal scans, with a high degree of certainty.

This paper provides a practical approach to the application of SPECT/CT image acquisition and its post-processing in order to accurately establish the diagnosis of cardiac amyloid infiltration.

It also emphasizes the importance of challenging the norms and re-evaluating diagnoses when improved and/or refined techniques illustrate shortcomings of current practice with SPECT only and planar imaging with H/CL measurements. Moving forward, the SPECT/CT fusion technique appears to be a highly efficient tool to avoid equivocal and/or false positive studies. The authors believe that this approach addresses problems in the diagnosis and treatment of cardiac amyloidosis, as it promotes timeous initiation of appropriate therapy where needed and prevents the use of unnecessary medication where it is not indicated.

The challenging imaging studies below demonstrate the application of this technique.

## CASE 1: SPECT versus SPECT/CT

**Case description**: 85-year-old female with paroxysmal atrial fibrillation and HFpEF.

**Figure 3:**
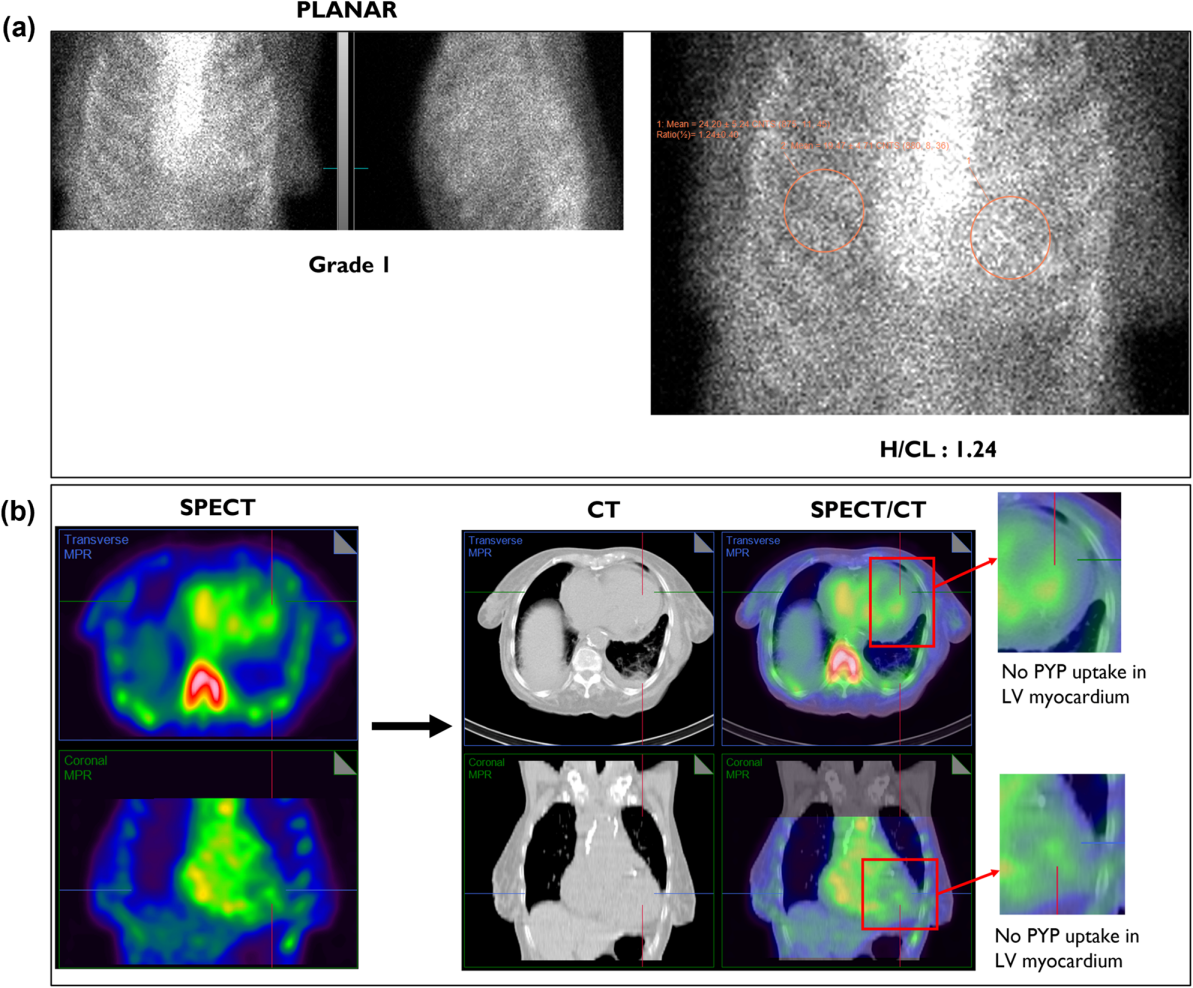
(A) Three-hour planar imaging shows Grade 1 visual tracer uptake and H/CL ratio of 1.24; interpreted as “equivocal for ATTR amyloidosis”.^5^ Visual assessment of planar images further demonstrates relatively less myocardial uptake compared to the ribs. (B) SPECT alone demonstrates an empty looking LV cavity, which is shown to be the thickened septal wall on the fused CT images. Also note that the inferior lateral border of the LV wall is not demonstrating any PYP uptake with SPECT/CT fusion. SPECT/CT reclassifies the case from equivocal to “not suggestive”

**PEARL: **Grade 1 visual tracer uptake and H/CL ratio of 1.24 (Figure 3a) and an empty looking LV cavity in SPECT classifies into “equivocal” as per the Expert Consensus Recommendations.^5^ However, when fusing SPECT with CT, it is clear that the empty looking LV cavity is in fact the thickened septum and that there is no PYP uptake in the LV myocardium (Figure 3b).

Planar imaging and H/CL ratio represent the summation of all the counts originating from the myocardium, the cardiac cavities, the ribcage, to the spine; however, they do not determine whether most of the counts originate from the amyloid infiltrated myocardium. H/CL ratio is derived directly from planar imaging, which does not discriminate location of the counts, i.e., it can't determine where the emitted counts originate from, hence providing only a total mean of overall counts in the drawn region of interest (ROI). This highlights the limitations of planar imaging and supports the notion that reliance on planar imaging can only be used if there is virtually no uptake in the cardiac region.

SPECT visual assessment alone is often not only insufficient but also misleading, as it leads to an incorrect interpretation of thickened septal wall as an empty looking LV cavity, and the LV cavity to appear as thickened inferolateral wall in axial images. However, SPECT/CT fusion confirms that there is no tracer uptake in the LV myocardium, changing the result from “equivocal” to “not suggestive" of ATTR amyloidosis.

***Key learning:*** Planar imaging and SPECT alone is not sufficient to confidently assess the LV myocardial uptake. SPECT/CT fusion offers precise anatomical referencing that allows an accurate determination of the origin of the counts.

## CASE 2: Thickened Septum

**Case description**: 61-year-old female known with ischemic cardiomyopathy and paroxysmal atrial fibrillation. Co-morbidities include diabetes, hypertension, chronic kidney disease, and obstructive sleep apnea.

**Figure 4:**
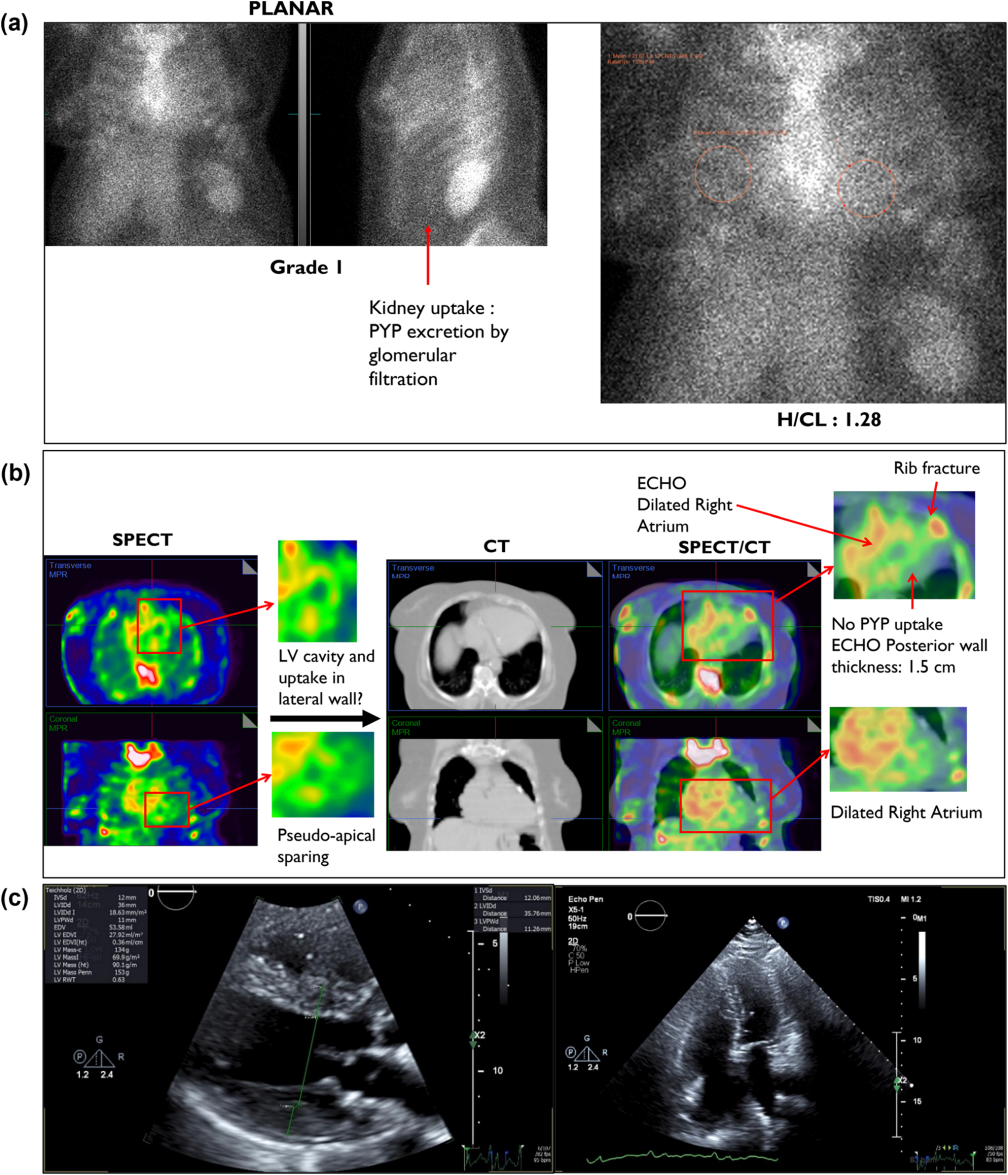
(**A**) Three-hour planar imaging showing Grade 1 tracer uptake and H/CL of 1.28; interpreted as “equivocal for ATTR amyloidosis”.^5^ Note the physiologic kidney uptake of PYP. (**B**) SPECT demonstrates empty looking LV cavity and a focal uptake in the rib on the right. There is also an *appearance* of apical sparing (pseudo-apical sparing). Note the rib fracture and enlarged RA and left atrium (LA). SPECT/CT demonstrates no PYP uptake in the LV myocardium, specifically in the thickened posterior wall. SPECT/CT reclassifies case from equivocal to “not suggestive.” (**C**) ECHO shows posterior wall thickness of 1.5 cm. Note dilated RA and LA

**PEARL: **H/CL ratio of 1.28 and Grade 1 tracer uptake by visual assessment (Figure 4a) classifies into “equivocal for ATTR amyloidosis”.^5^ SPECT images demonstrates an empty looking LV cavity with mild PYP uptake in the lateral wall and the appearance of apical sparing (Figure 4b).

However, CT and SPECT/CT images clearly shows a dilated right atrium (RA) which correlates with the findings of the ECHO which shows an enlarged RA and posterior wall thickness of 1.5 cm (Figure 4c). SPECT/CT images also demonstrate no tracer uptake in the LV myocardium, changing the classification from “equivocal” to “not suggestive.” In addition, mitral valve calcification and pacemaker leads are noted in CT and SPECT/CT images (Figure 4b). This case highlights how a non-tracer avid but thickened LV wall, specifically the septum, can display as LV cavity (no tracer uptake) and the distal apical septum displaying as apical sparing.

***Key learnings:*** Increased blood pool activity in any of the chambers (atria, right, and left ventricles) will lead to elevation of the H/CL ratio in patients with reduced cardiac output, pulmonary hypertension, or LV dysfunction. The persistence of the blood pool activity at 3 hours would be falsely interpreted as increased H/CL ratio. However, by utilizing SPECT/CT fusion, a direct visualization of the endocardial borders and the myocardium will immediately highlight the fact that the uptake is in the blood pool and not in the myocardium.

## CASE 3: Pseudo-apical sparing

**Case description**: 68-year-old female known with coronary artery disease and HFpEF. Co-morbidities include diabetes mellitus and hypertension.

**Figure 5:**
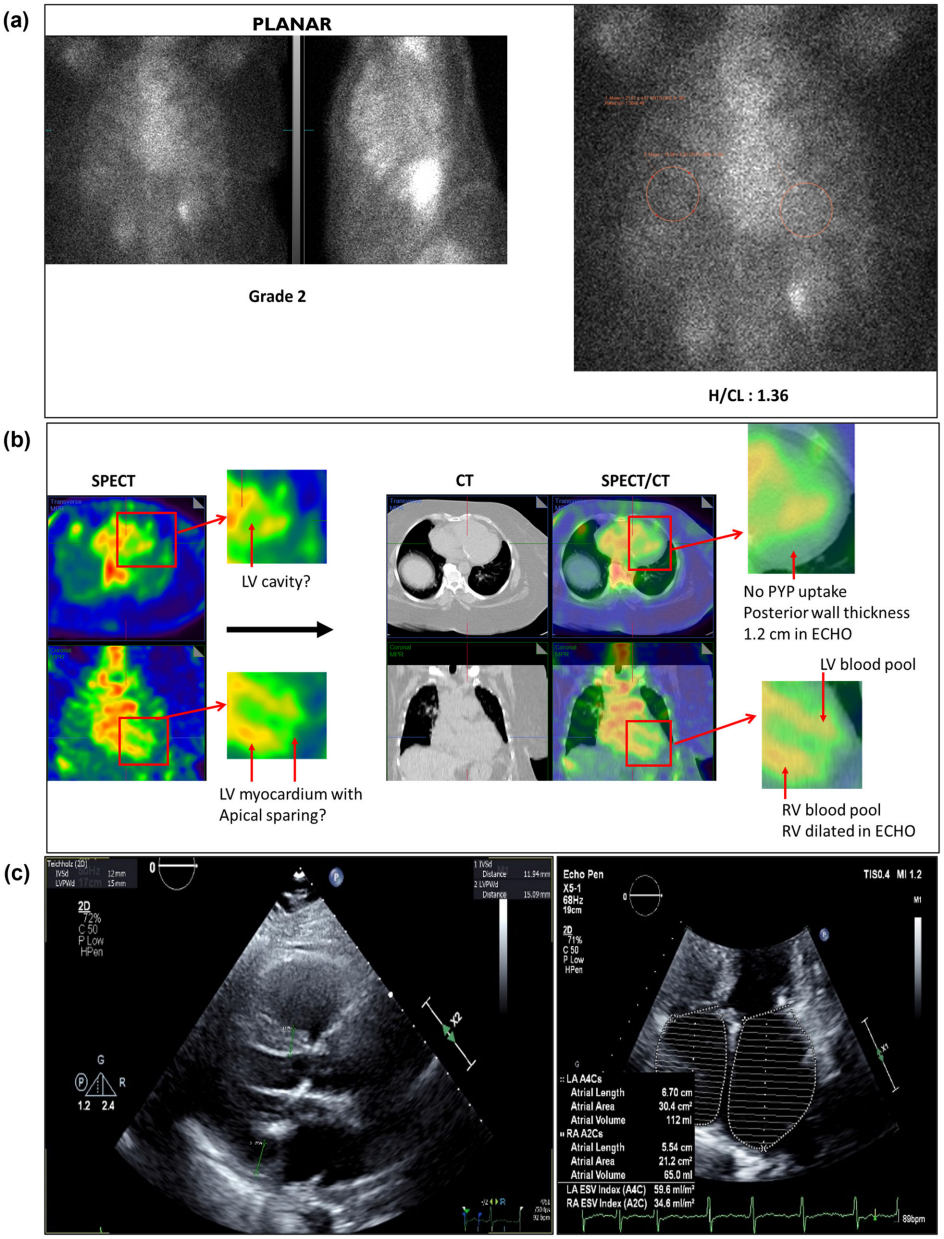
Pseudo-apical sparing (**A**) Three-hour planar imaging shows Grade 2 tracer uptake and H/CL ratio of 1.36; interpreted as “strongly suggestive of ATTR amyloidosis”.^5^ (**B**) SPECT appears to demonstrate LV wall tracer uptake, sparing the apex. SPECT/CT shows no PYP uptake in the myocardium, but rather confirms LV and RV blood pool. Note dilated RV and thickened posterior wall (1.2 cm). SPECT/CT reclassifies case from strongly suggestive to not suggestive. (**C**) Four chamber ECHO clearly indicates enlarged RA and RV. Posterior wall is 1.2 cm, septum is 1.2 cm

**PEARL:** H/CL ratio of 1.36 and Grade 2 tracer uptake by visual assessment (Figure 5a) and an empty LV cavity with PYP uptake in the lateral wall in SPECT (Figure 5b) is interpreted as “strongly suggestive” as per the Expert Consensus Recommendations.^5^ Based on the authors’ experience, pseudo-apical sparing is an additional new deceptive finding if SPECT only imaging is utilized.

**Figure 6:**
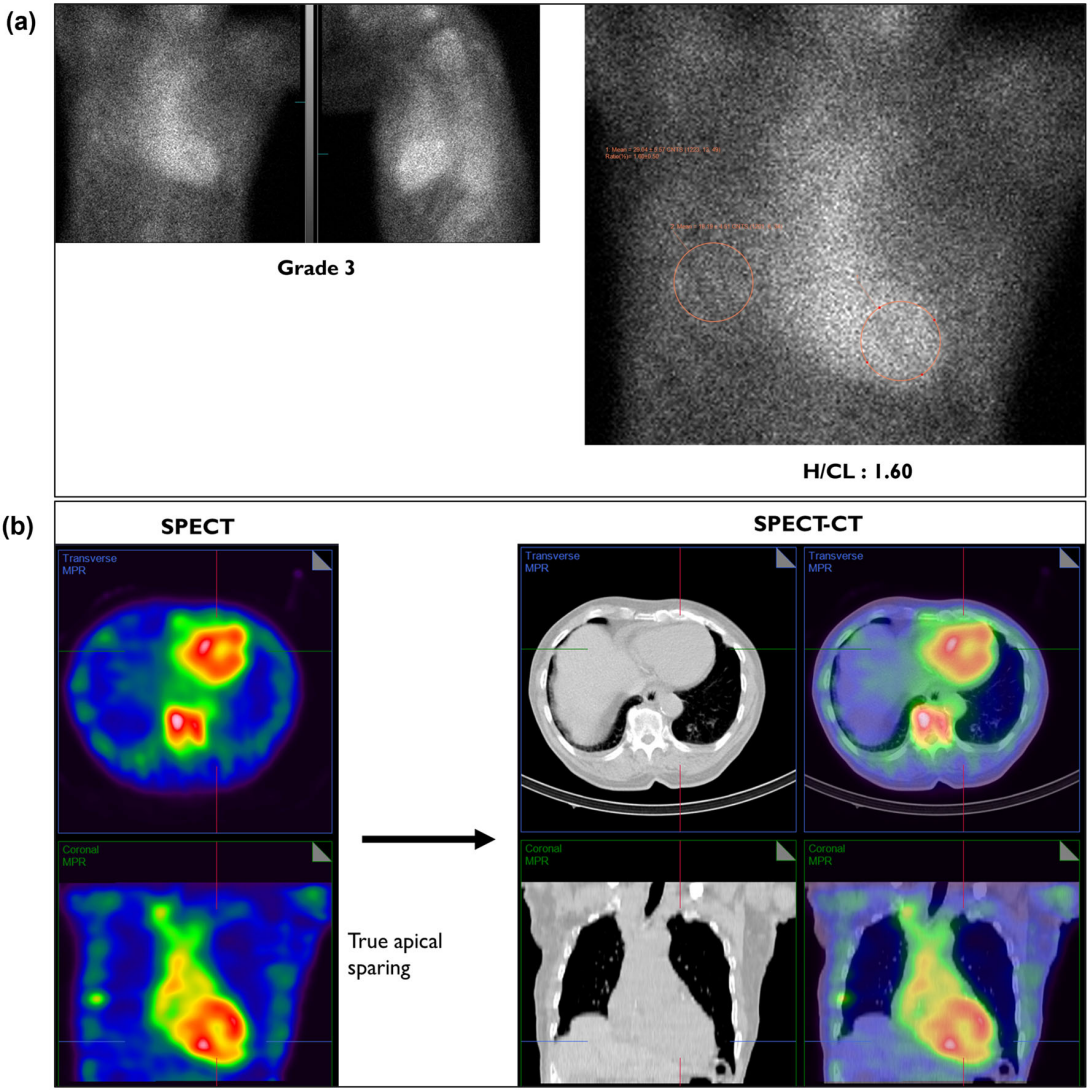
True Apical sparing (**A**) Three-hour planar imaging shows Grade 3 tracer uptake and H/CL ratio of 1.60; interpreted as “strongly suggestive of ATTR amyloidosis”.^5^ **B** Apical sparing is evident in the SPECT as well as SPECT/CT. Note thickened LV myocardium in SPECT and SPECT/CT images. SPECT/CT fusion confirms classification as strongly suggestive of ATTR amyloidosis (see Appendix A, Case 7)

Pulmonary hypertension and dilated right sides (RA and RV) may create this pattern of SPECT images (Figure 5b) that appear to indicate an empty looking LV cavity with uptake in the LV myocardium and pseudo-apical sparing. However, four chamber ECHO (Figure 5c) clearly demonstrates a dilated RV and RA with posterior wall thickness of 1.2 cm. This is validated by fusing CT and SPECT, where SPECT/CT images show an enlarged RV blood pool and RA blood pool with no tracer uptake in the LV myocardium (Figure 5b), changing the interpretation from “strongly suggestive of ATTR amyloidosis” to “not suggestive.” Figure 6 represents a case with true apical sparing for comparison (see Appendix A: Case 7).

***Key learnings: *** Enlarged RV and/or RA may create pseudo-apical sparing and confound findings of SPECT images, leading to an incorrect interpretation of the findings as “strongly suggestive” (see Figure 4b and Appendix A: Case 7).

## CASE 4: Example of accurate SPECT and CT registration

**Case description**: 73-year-old male known with ischemic cardiomyopathy. Co-morbidities include diabetes mellitus, hypertension, and chronic kidney disease.

**Figure 7:**
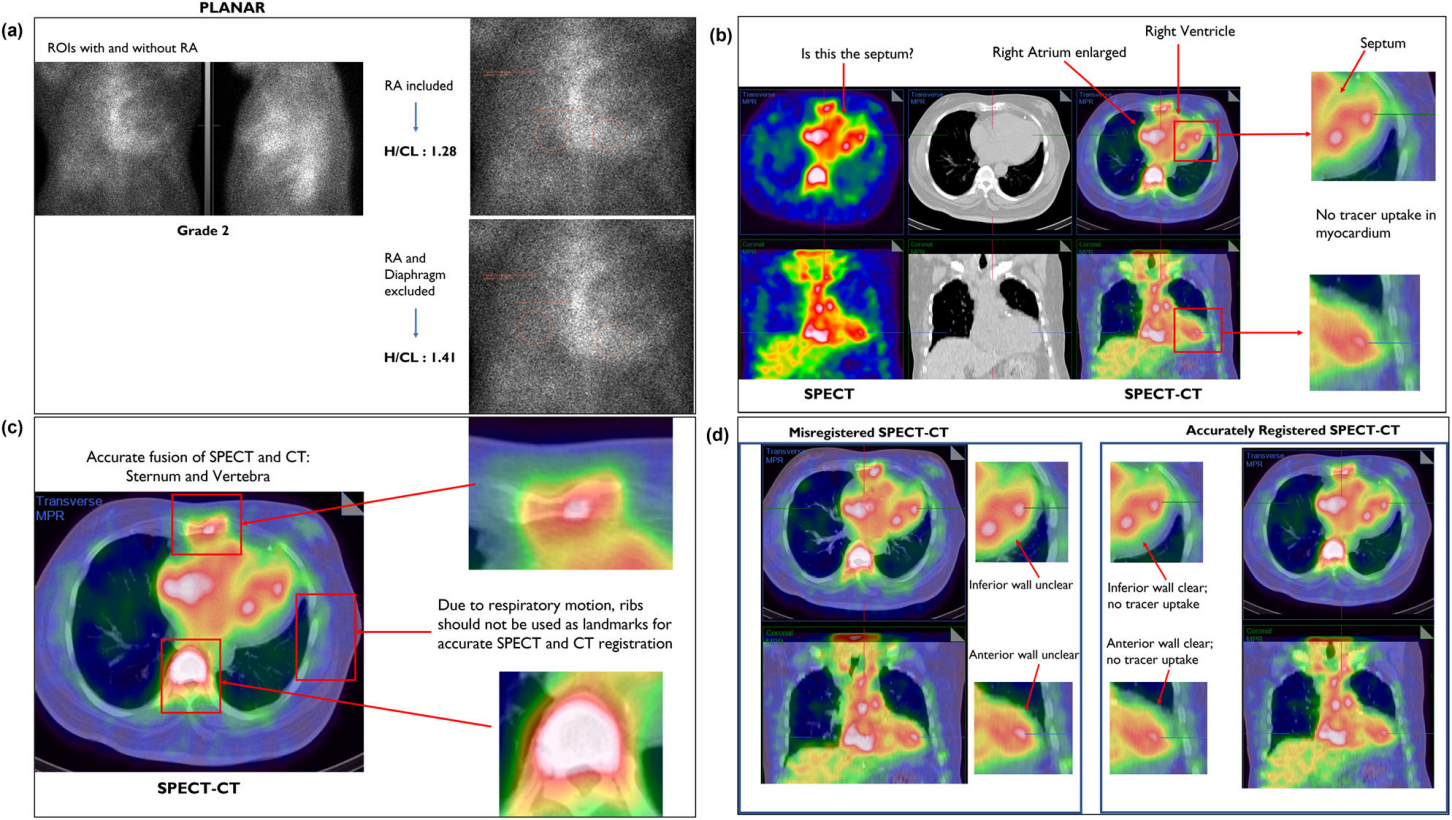
**A** Three-hour planar imaging demonstrates difference in H/CL ratio calculation when enlarged RA is included in ROI *vs* when RA and diaphragm is excluded. Note lower H/CL ratio when RA is included. **B** SPECT fails to clearly distinguish between the septum and the enlarged RA and RV. SPECT/CT images clearly show enlarged RA and distinguish septum, RA and RV. SPECT/CT images demonstrates no PYP uptake in the myocardium, changing the classification from strongly suggestive to not suggestive. **C** Sternum and vertebral body are reference landmarks to accurately fuse the SPECT and CT images. **D** On the left, SPECT and CT images are inaccurately fused. On the right, SPECT and CT images are precisely fused. With precise registration (on the right), non-tracer avid infero-posterial wall (axial slice, right upper corner) and anterior wall (coronal slice, right lower corner) increase in thickness and are clearly distinguishable as non-tracer avid regions. In the misregistered SPECT/CT images (on the left), the inferior wall appears approximately 3–4 mm; with accurate registration it appears approximately 1.5 mm. Note: the sternum and vertebral body are reference landmarks to be used for accurate registration of SPECT and CT images, which are less effected by the significant inherent respiratory motion. Ribs should not be used as reference landmark given much greater respiratory movement

**PEARLS:**SPECT/CT images demonstrate that the RA is markedly enlarged compared to the RV, which is not clear in the planar images. For H/CL calculation, without knowing the RA is enlarged, the circle is placed on part of the RA which lowers the H/CL calculation (Figure 7a). Furthermore, visual grading of lateral planar images is scored as Grade 2 (myocardial uptake equal to the ribs), as the ribs cannot be visually traced individually. With proper placement of contralateral lung ROI (by excluding enlarged RA), H/CL ratio changes to 1.41, which is “strongly suggestive” and concordant with the visual assessment of Grade 2.

When SPECT only images are used, defining the location of the septum is the most challenging, as such a thickened septum may be misinterpreted as LV cavity. SPECT images demonstrate an empty looking LV cavity surrounded by LV myocardium with PYP uptake (Figure 7b).

Inaccurate SPECT/CT registration or misregistration can add confusion and error, hence precision is needed by utilizing reference anatomical landmarks (i.e., sternum and vertebral body) (Figure 7d). Given a higher degree of respiratory motion, the ribs should not be used as a reference landmark for SPECT and CT registration; rather the sternum and vertebral body should always be used to accurately fuse the SPECT and CT images (Figure 7c and 7d).

The process of reconstruction and post-processing of the images are described in detail in “reconstruction and post-processing of the images” in the Supplementary Material.

***Key learnings: The detailed anatomical referencing provided with SPECT/CT fusion has the unique benefit of accurate localization of the septum. Incorrect fusion technique may create difficulty in correctly identifying the septum and the posterior-lateral border of the LV myocardium. Localization of the septum helps to differentiate the boundaries of myocardial vs blood pool uptake.***

## CASE 5: Three-hours vs six-hours imaging

**Figure 8:**
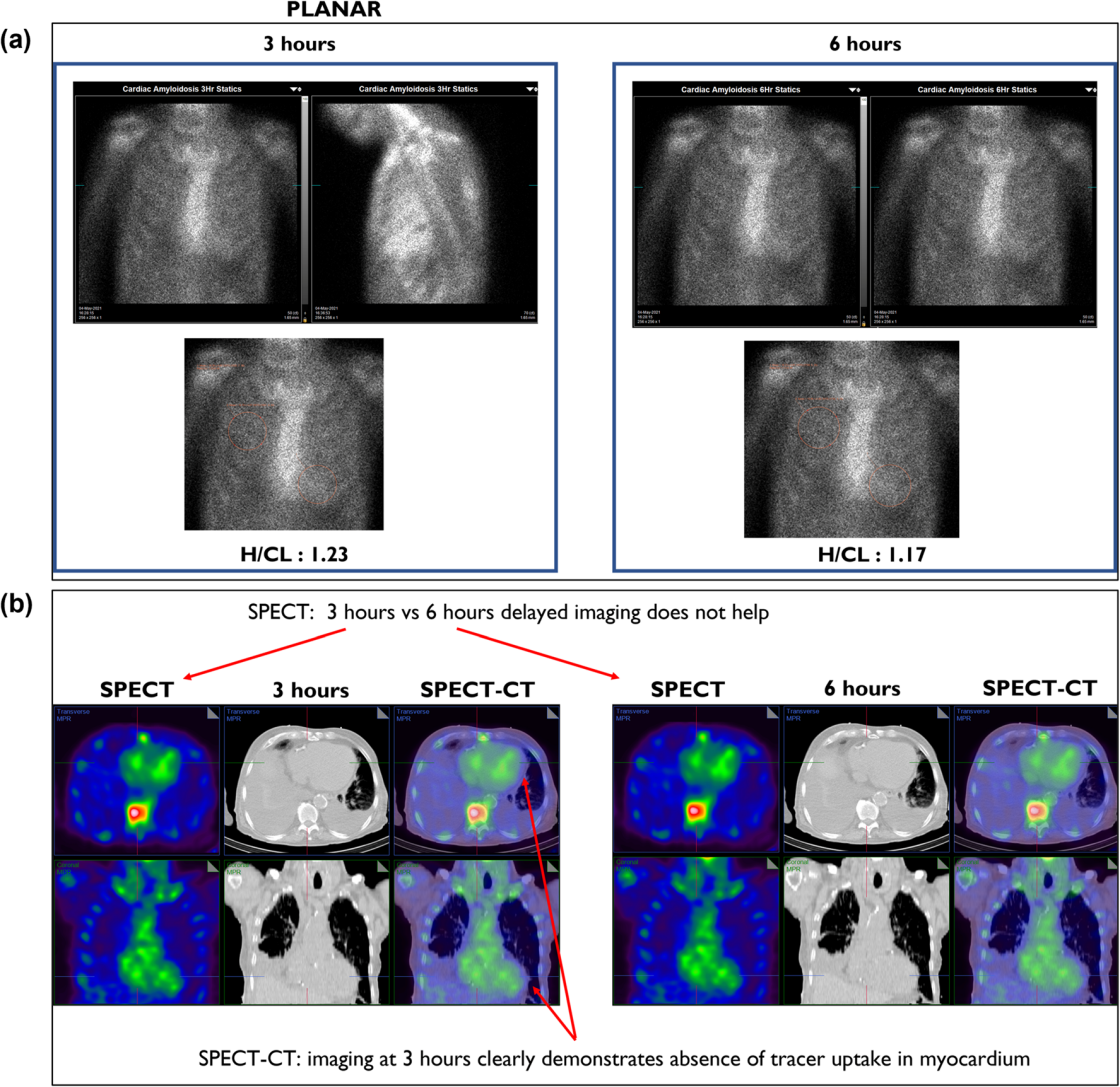
(**A**) Three-hours planar imaging *vs* six-hour planar imaging: H/CL ratio of 1.23 and 1.17 respectively, Grade 1 uptake in both planar images, demonstrating that 6 h delayed imaging does not add value. **B** SPECT/CT at three hours demonstrates absence of myocardial PYP uptake. SPECT/CT reclassifies case from equivocal to not suggestive

**Case description**: 88-year-old male known with ischemic cardiomyopathy and severe aortic stenosis post transcatheter aortic valve implantation (TAVI). Co-morbidities include diabetes mellitus, hypertension, and chronic kidney disease (Figure 8).

**PEARL:** SPECT/CT imaging at three hours is not suggestive; rules out equivocal interpretation with SPECT only imaging, with no added benefit of six hours SPECT only imaging.

***Key learning: In the setting of equivocal interpretation at three hours SPECT only imaging, six hours SPECT only imaging does not have any additional diagnostic value. In contrast, SPECT/CT imaging is not suggestive at three hours with a definitive interpretation.***

## New Knowledge Gained

SPECT/CT fusion technique significantly improves the accuracy of interpretation of cardiac amyloid imaging with one of the most commonly used bone seeking radiotracers (PYP) by eliminating equivocal, and markedly reducing false positive results, when compared to SPECT only imaging.

## Study limitations

As patients are referred to our center from practitioners outside of our facility, many of the patients in our cohort were low pre-test probability patients. This explains the low yield of true positive cases (*n* = 11; 6%). It is understood that in the USA, SPECT/CT cameras are used in large Academic institutions; however, in private practice, SPECT only cameras are primarily utilized, and the imaging is performed in outpatient cardiology offices. In the Middle-East, North Africa, and Asia Pacific for example, PYP scans are performed in institutional hospitals; there is no private practice cardiology or stand-alone cameras. Therefore, as resources between countries and centers within the same country differ, it may not be possible for all practitioners or centers to perform SPECT/CT fusion as part of their standard practice. While we strongly believe that SPECT/CT fusion is a valuable tool, we recognize that the availability of the technology will have an impact on its utilization.

## Conclusion

This paper demonstrates the significant impact of the SPECT/CT fusion technique in improving the diagnostic accuracy of PYP imaging, given SPECT only imaging may not always adequately differentiate between LV cavity tracer uptake and true myocardial uptake. SPECT/CT fusion significantly reduces equivocal and false positive results, and also has the ability to reduce the number of false positive results in low pre-test probability patients. We propose that where the resources are available, SPECT/CT fusion should be the standard of care imaging due to its highly effective impact in sensitivity and specificity and overall diagnostic accuracy. Large multi-center studies will be helpful in confirming our data.

### Supplementary Information

Below is the link to the electronic supplementary material.

Supplementary file1 (DOCX 1457 kb)

## Abbreviations

99mTc-PYP: Technethium-99 m pyrophosphate
AL: Amyloidosis, immunoglobulin light chain amyloidosis
ASNC: American Society of Nuclear Cardiology
ATTR: Amyloidosis, amyloid transthyretin amyloidosis
ATTR-CM: Transthyretin amyloid cardiomyopathy
CCAD: Cleveland Clinic Abu Dhabi
CT: Computed tomography
ECHO: Echocardiography
H/CL: Heart to contralateral lung
HFpEF: Heart failure with preserved ejection fraction
LV: Left ventricle
PYP: Pyrophosphate
RA: Right atrium
ROI: Region of interest
RV: Right ventricle
SPECT: Single-photon emission computed tomography
