# Supplementary material for: Attacking the Achilles heel of cardiac amyloid nuclear scintigraphy: How to reduce equivocal and false positive studies
Source: J Nucl Cardiol. 2023 Mar 1;30(5):1922–34. doi: 10.1007/s12350-023-03214-6 (PMC10558365; doi:10.1007/s12350-023-03214-6)
Supplement: Supplementary file 1 — Supplementary file1 (DOCX 1457 kb) [file 12350_2023_3214_MOESM1_ESM.docx]

**APPENDIX A**

**Manuscript: Attacking the Achilles heel of Cardiac Amyloid Nuclear Scintigraphy: How to eliminate equivocal and false positive studies**

**Authors:** Zainab Al Taha^a^, MD; Deniz Alibazoglu^b^, MPH, Hani Sabbour^c^, MD; Ingy Romany^d^, Haluk Alibazoglu^e^, MD, PhD, Sabahat Bokhari^f^, MD

**Affiliations:**

^a^Sheikh Shakhboot Medical City, Abu Dhabi, United Arab Emirates; ^b^Northwestern University, Chicago, IL, United States; ^c^Cleveland Clinic Abu Dhabi, Abu Dhabi, United Arab Emirates; Warren Alpert School of Medicine, Brown University, Providence, RI, United States; ^d^Pfizer Gulf FZ-LLC, Dubai, United Arab Emirates; ^e^Cleveland Clinic Abu Dhabi, Abu Dhabi, United Arab Emirates; ^f^ Robert Wood Johnson Medical School – Rutgers University, New Brunswick, NJ, USA

**Corresponding author:** Dr Hani Sabbour

**Telephone / fax number:** +9715209115968

**E-mail address:** hanisabbour1@icloud.com

**Address:** Cleveland Clinic Abu Dhabi, Abu Dhabi, United Arab Emirates

**METHODS**

**Reconstruction and post-processing of the images**

Planar image acquisition was completed in anterior and lateral projections 3 hours following injection of 20 mCi (+/- 10%) ^99m^TC-PYP. Acquisition parameters: Low Energy High Resolution Collimator, 140 keV(15%) energy window, 256 x 256 matrix, 1.45 zoom, 180-degree detector configuration and 750 kcts. CT attenuation correction: 1 mCi Thallium 201: approximately 5 mSv. Low dose Attenuation Correction CT: <1 mSv (1-4).

Following planar image acquisition, SPECT/CT images of the thorax were acquired using a 2 slice SPECT/CT scanner (Symbia T, Siemens Medical Solutions, USA). SPECT acquisition parameters: Low Energy High Resolution Collimator, 140 keV (15%) energy window, 128 x 128 matrix, 1.45 zoom, 180-degree detector configuration, non-circular continuous orbit, 32 stops, 23 seconds per stop. CT acquisition parameters: (CARE Dose 4D) were: 130 kV, 1.5 Pitch, 5.0 mm slice, 2 x 2.5 acquisition, 0.8 sec rotation time, craniocaudal direction.

Image reconstruction was performed on Siemens Syngo application by utilizing a custom amyloid SPECT/CT reconstruction protocol in the Symbia T acquisition workstation. Following quality control checks for motion, full field of view reconstruction was completed by utilizing Flash 3D, 4 iterations, 8 subset, Gaussian filter with 8.4 mm FWHM. After automated image fusion, attenuation corrected file with associated mu map of fused images along with static planar images were transferred to Siemens Syngo.via platform for postprocessing by Corridor 4DM (Invia Medical Imaging Solutions, Ann Arbor, MI, USA) application. For SPECT CT image analysis, SPECT AC reconstructed file is selected as the object and CT mediastinal window b30s file is selected as the target in SPECT/CT fusion tab of Corridor 4DM and displayed in axial, coronal and sagittal planes. All of the fused SPECT/CT reconstructed images are displayed in spectrum colour scale. Grey scale was used for review of CT images in appropriate windows for incidental findings (i.e., effusions, calcifications, metallic objects).

For accurate registration of the SPECT and CT images, sternum and vertebral body were used as anatomical landmarks to achieve precision in fusion of both data sets. Due to greater respiratory motion, ribs should not be used as anatomical landmarks.

**DISUCSSION**

**Pseudo-apical sparing *vs* True Apical sparing**

**Case 6: Pseudo-apical sparing with no right atrial or right ventricular enlargement**

**Case description**: 66-year-old female with HFpEF. Co-morbidities include diabetes mellitus and hypertension.


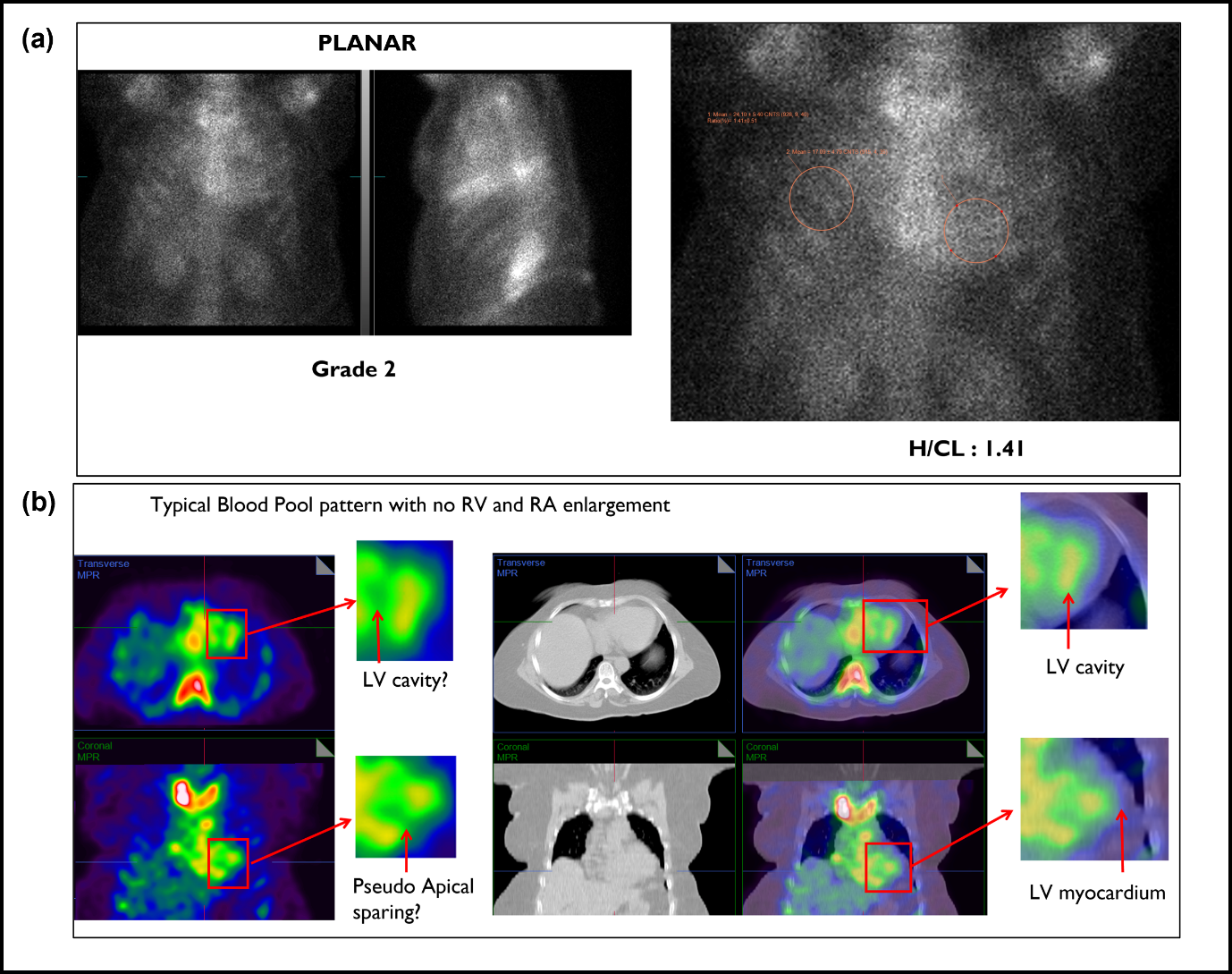


**Fig. 9 (a)** Three-hour planar imaging shows Grade 2 tracer uptake and H/CL ratio of 1.41; interpreted as “strongly suggestive of ATTR amyloidosis”(5). **(b)** SPECT shows empty looking LV cavity with tracer uptake in lateral wall. However, SPECT/CT demonstrates thickened LV myocardium with no PYP uptake. Note the typical blood pool pattern with no right atrial and right ventricular enlargement. SPECT/CT fusion reclassifies case from strongly suggestive to not suggestive.

**PEARLS:** H/CL ratio of 1.41 and Grade 2 tracer uptake by visual assessment and an empty looking LV cavity with tracer uptake in the lateral wall in SPECT classifies into “strongly suggestive” as per the Expert Consensus Recommendations(5). SPECT images demonstrate an appearance of apical sparing (Pseudo-apical sparing) in axial and coronal images. ECHO demonstrates normal sized biventricular cavities and normal wall thickness. SPECT/CT images demonstrates no tracer uptake in the LV myocardium, changing the classification from “suggestive” to “not suggestive”. This case further highlights that even without dilated right sides and pulmonary hypertension, SPECT only imaging may result incorrect strongly suggestive interpretation.

***Key learnings:*** *In more advanced cases of HFpEF, with enlargement of both sides and associated blood pooling, SPECT images may demonstrate pseudo-apical sparing (see Case 3 in manuscript and Case 7 below). However, in this case, which is a less advanced stage of HFpEF, pseudo-apical sparing is still apparent in SPECT images in which tracer uptake is primarily in the LV cavity, without enlargement of right sides and blood pooling.*

**Case 7: True apical sparing**

**Case description**: 77-year-old male known with HFpEF

**PEARL:** Visual assessment of the planar images (Grade 3 tracer uptake and H/CL ratio of 1.60) **(Fig. 5a)** classifies this case as “strongly suggestive of ATTR amyloidosis”(5). SPECT and SPECT/CT fusion confirms this a true positive case, as it clearly shows LV myocardium PYP uptake, with less counts in the cavity, i.e., true apical sparing **(Fig.5b)**. While apical sparing has been well characterized with ECHO with reduced global longitudinal strains and apical sparing on strain imaging, it is also described in nuclear imaging as reduced uptake in the apical myocardium due to likely reduced deposition of the amyloid fibrils(6).

***Key learning:*** *In SPECT only imaging, True apical sparing can be very similar in appearance to pseudo-apical sparing, as shown in Cases 2, 3 in the manuscript and Case 6 above.*

**References**

1. Cerqueira MD, Allman KC, Ficaro EP, Hansen CL, Nichols KJ, Thompson RC, et al. Recommendations for reducing radiation exposure in myocardial perfusion imaging. J Nucl Cardiol. 2010;17(4):709-18.

2. Hendel RC, Berman DS, Carli MFD, Heidenreich PA, Henkin RE, Pellikka PA, et al. ACCF/ASNC/ACR/AHA/ASE/SCCT/SCMR/SNM 2009 Appropriate Use Criteria for Cardiac Radionuclide Imaging. Journal of the American College of Cardiology. 2009;53(23):2201-29.

3. Depuey E, Mahmarian J, Miller T, Einstein A, Hansen C, Holly T, et al. Patient-centered imaging. Journal of nuclear cardiology : official publication of the American Society of Nuclear Cardiology. 2012;19:185-215.

4. DePuey EG. Advances in SPECT camera software and hardware: currently available and new on the horizon. J Nucl Cardiol. 2012;19(3):551-81; quiz 85.

5. Dorbala S, Ando Y, Bokhari S, Dispenzieri A, Falk RH, Ferrari VA, et al. Addendum to ASNC/AHA/ASE/EANM/HFSA/ISA/SCMR/SNMMI Expert Consensus Recommendations for Multimodality Imaging in Cardiac Amyloidosis: Part 1 of 2-Evidence Base and Standardized Methods of Imaging. J Card Fail. 2021.

6. Sperry BW, Vranian MN, Tower-Rader A, Hachamovitch R, Hanna M, Brunken R, et al. Regional Variation in Technetium Pyrophosphate Uptake in Transthyretin Cardiac Amyloidosis and Impact on Mortality. JACC Cardiovasc Imaging. 2018;11(2 Pt 1):234-42.
